# DLC1 inhibits colon adenocarcinoma cell migration by promoting secretion of the neurotrophic factor MANF

**DOI:** 10.3389/fonc.2022.900166

**Published:** 2022-09-14

**Authors:** Yi-Min Chu, Ying Xu, Xiu-Qun Zou, Feng-Li Zhou, Yu-Liang Deng, Yu-Tong Han, Ji Li, Da-Ming Yang, Hai-Xia Peng

**Affiliations:** ^1^ Digestive Endoscopy Center, Tongren Hospital, Shanghai Jiao Tong University School of Medicine, Shanghai, China; ^2^ Department of Biochemistry and Molecular Cellular Biology, Shanghai Jiao Tong University School of Medicine, Shanghai, China; ^3^ Shanghai Center for Systems Biomedicine, Key Laboratory of Systems Biomedicine (Ministry of Education), Shanghai Jiao Tong University, Shanghai, China

**Keywords:** colorectal cancer (CRC), deleted in liver cancer-1 (DLC1), conditioned medium (CM), mesencephalic astrocyte-derived neurotrophic factor (MANF), migration

## Abstract

DLC1 (deleted in liver cancer-1) is downregulated or deleted in colorectal cancer (CRC) tissues and functions as a potent tumor suppressor, but the underlying molecular mechanism remains elusive. We found that the conditioned medium (CM) collected from DLC1-overexpressed SW1116 cells inhibited the migration of colon adenocarcinoma cells HCT116 and SW1116, but had no effect on proliferation, which suggested DLC1-mediated secretory components containing a specific inhibitor for colon adenocarcinoma cell migration. Analysis by mass spectrometry identified mesencephalic astrocyte-derived neurotrophic factor (MANF) as a candidate. More importantly, exogenous MANF significantly inhibited the migration of colon adenocarcinoma cells HCT116 and SW1116, but did not affect proliferation. Mechanistically, DLC1 reduced the retention of MANF in ER by competing the interaction between MANF and GRP78. Taken together, these data provided new insights into the suppressive effects of DLC1 on CRC, and revealed the potential of MANF in the treatment of CRC.

## Introduction

The incidence and mortality of colorectal cancer (CRC) have been rapidly rising in recent years (1). According to estimates from Global Cancer Statistics 2018, CRC was the third most commonly diagnosed cancer, and was expected to rank as the second leading cause of death in the world. Although the treatment had been optimized, the mortality rate remained high, which was mainly attributed to metastatic progression (2).

Deleted in liver cancer-1 (DLC1) has been accepted as a crucial tumor suppressor in extensive human cancers since its first identification in 1998 (3), and it is shown to be mutated as often as p53 in cancers (4). It encodes a RhoGTP activating protein (RhoGAP) and impedes GTPases like RhoA, RhoB, RhoC, and Cdc42 by prompting the hydrolysis active GTP-bound state (5, 6). These Rho GTPases are dominant regulators of cytoskeleton dynamics and focal adhesion (FA) formation (6). DLC1 was reported to suppress parathyroid hormone-like hormone (PTHLH) transcription and secretion through Rho-TGF-β crosstalk, leading to attenuate osteoclast maturation and reduce breast cancer bone metastasis (7). This study uncovered a cytoskeleton-independent role of DLC1 in cancer metastasis, but it depended on RhoGAP function yet.

Mesencephalic astrocyte-derived neurotrophic factor (MANF) is a secretory protein, which is highly conserved in all species (8). For the last two decades, MANF has been focused as a cytoprotective factor in rodent models of Parkinson’s disease, cerebral ischemia, spinocerebellar ataxia, and myocardial infarction (8). MANF knockout mice suffering from diabetes and growth defect indicate the functional diversity of this protein (8, 9). Pancreas-specific deletion of MANF mice demonstrated activation of unfolded protein response (UPR) genes and sustained stimulation of the endoplasmic reticulum (ER) stress marker, which suggested the strong association between MANF and ER stress (9). The latest study linked MANF to hepatocellular carcinoma *via* ER stress and inflammation (10).

In the present study, we reported that high expression of DLC1 promoted MANF secretion and the exogenous MANF could significantly inhibit the migration of colon adenocarcinoma cells without affecting cell proliferation, which indicated that MANF was a key molecule in the mechanism of DLC1 suppression of CRC metastasis.

## Materials and methods

### Plasmids

The cloning strategy was described before (11). The human DLC1 (NM_006094.5) and GRP78 (NM_005347.5) cDNA were subcloned into pcDNA3.1 vector or pcDH vector and the Flag-tag or HA-tag was added at the N-terminus of DLC1 protein and at the C-terminus of GRP78 protein. The human MANF (NM_006010.6) cDNA was subcloned into pcDH-vector and the Flag-tag was added behind the signal peptide of MANF protein. The pGIPZ-shMANF plasmid was from the DNA library of Shanghai Jiaotong University School of Medicine and the sense sequence is listed in **Table 1**.

**Table 1:** Primer list.

	Primers	Sequences
Primer for qRT-PCR	DLC1 Forward	GGACACCATGATCCTAACAC
	DLC1 Reverse	AGTCCATTTGCCACTGATGG
	MANF Forward	TGTCACATTCTCACCAGCCACT
	MANF Reverse	CAGGTCGATCTGCTTGTCATACTT
Sense sequences	MANF shRNA	AGCCAGATATGTGAGCTTA

### Cell culture, transfection, and lentivirus infection

HEK-293T and human colon adenocarcinoma cell lines SW1116 and HCT116 were obtained from the American Type Culture Collection (ATCC) and were cultured at 37°C in 5% CO_2_ according to established protocols (12). Lipofectamine 3000 (Invitrogen, USA) was used to deliver the plasmids into cells. Supernatants containing lentivirus were packaged in HEK-293T cells, and infected cells were assessed to detect the effect by quantitative real-time PCR (qRT-PCR) and Western blot.

### Quantitative real-time PCR

Total RNA was extracted from cells with TRIzol reagent (Invitrogen, USA) following the manufacturer’s protocol. Reverse transcription was performed using the Transcriptor First Strand cDNA Synthesis Kit (Roche, Switzerland). The quantification and analysis of target mRNA were performed using qRT-PCR (SYBR Green, ABI, USA) with specific primers (**Table 1**).

### Co-immunoprecipitation, Western blot, and immunofluorescence

Co-IP, Western blot, and immunofluorescence (IF) assays were performed as described previously (11, 13). Antibodies used in these assays were as follows: MANF (10869-1-AP) antibody was purchased from Proteintech (P.R.C). Anti-Flag M2 (M8823) Magnetic Beads and anti-Flag M2 (B3111) antibody were purchased from Sigma-Aldrich (USA). HA-tag (#3274) antibody was purchased from Cell Signaling Technology (USA). GRP78 (sc-13539), β-actin (sc-81178) antibody were purchased from Santa Cruz Biotechnology (USA). The secondary antibody (A-11029, A-11077) used in IF was purchased from Thermo Scientific (USA).

### Conditioned medium collection

Stably overexpressed DLC1 SW1116 cells were cultured to 70%–80% confluency. The medium was replaced with a serum-free medium. After 48 h, the conditioned medium (CM) was collected, centrifuged at 15,000 rpm for 1 h, and filtered using a 0.22-μm filter (Millipore, USA) and concentrated at 4,500 rpm with Amicon Ultra-15 centrifugal filter devices (10 kDa, Millipore, USA). The concentration CM was stored at −80°C till further usage.

### Cell proliferation and migration assessment

Cell proliferation assay was examined using a CCK-8 kit (Dojindo, Japan) and cell migration was assessed with transwell assay (24 well, 8.0-μm pore membranes, Corning, USA) as described previously (12). We added CM or recombinant human MANF protein (ACE87065, R&D Systems, USA) into wild-type cell culture medium in proliferation assay, while we used CM or recombinant MANF protein into the top chamber in the migration assay.

### Mass spectrometry analysis

The samples to be tested were separated on an SDS-PAGE gel and silver stained. The specific band was cut and analyzed by Shanghai Hoogen Biotech Company (Shanghai, China) (14). Concisely, protein residues were digested by trypsin (Promega, USA) in 37°C for 16–18 h, and then the products were separated by high-performance liquid chromatography (HPLC). Q Exactive mass spectrometer (Thermo Scientific, USA) was used for analysis. The raw data were processed with Mascot 2.2 software. The proteins were identified by seeking against the UniProt database.

### ELISA

Remove particulates by centrifugation and assay immediately or aliquot and store samples at −80°C. In order to measure the concentration of MANF in the samples, the MANF ELISA Kit from Sino Best Biological Technology Co. (Shanghai, China) includes a set of calibration standards. The calibration standards are assayed at the same time as the samples and allow the operator to produce a standard curve of Optical Density versus MANF concentration. The concentration of MANF in the samples is then measured at 450 nm using a spectrophotometer (Thermo Scientific, USA).

### Statistical analysis

Each experiment was performed in triplicate. Statistical analysis was conducted using Student’s two-tailed Exact *t*-test to compare, with *p*-values < 0.05 as statistically significant. All statistical tests were performed using SPSS software (SPSS 13).

## Results

### The conditioned medium of DLC1-overexpressed SW1116 cells reduced migration of wild-type colon adenocarcinoma cells

**Figure 1:**
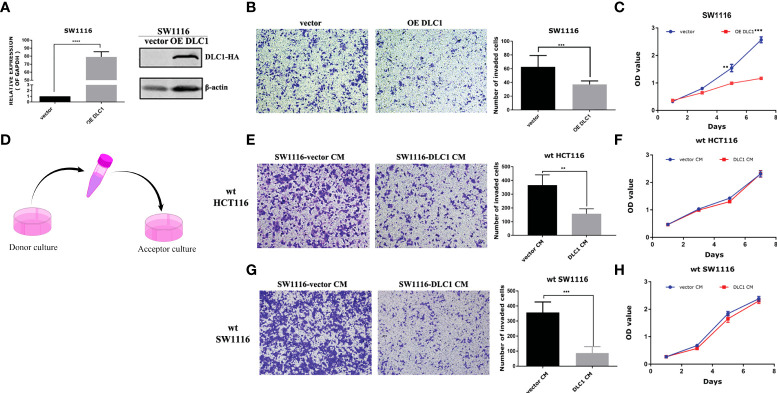
Culture medium of DLC1-overexpressed SW1116 cells inhibited the migration of wild-type colon adenocarcinoma cells. **(A)** We established a stable colon adenocarcinoma cell line with overexpression of DLC1 in SW1116 and confirmed by qRT-PCR and Western blot assays. The vector group cells were infected with non-target control virus. ***p < 0.001, compared with the vector group. **(B)** Overexpressed DLC1 inhibited migration of SW1116. ***p < 0.001, compared with the vector group. **(C)** Overexpressed DLC1 inhibited proliferation of SW1116. **p < 0.01, ***p < 0.001, compared with the vector group. **(D)** The way we collected and concentrated DLC1-overexpressed SW1116 cells’ CM to culture wild-type colon cell lines SW1116 By Figdraw and HCT116. Wild-type colon adenocarcinoma cell lines SW1116 **(E)** and HCT116 **(G)** cultured by DLC1-overexpressed CM (1 μg/ml) resulted in a significant reduction in cell migration. **p < 0.01, ***p < 0.001, compared with the vector group. SW1116 **(F)** and HCT116 **(H)** cultured by DLC1-overexpressed CM (2 μg/ml) had no changes in cell proliferation as compared with vector control CM.

DLC1 was consistently downregulated or absent in various malignant tumors (15), including CRC (**Figure S1**). We established stable colon adenocarcinoma cell line with overexpression of DLC1 in SW1116 *via* lentivirus infection (**Figure 1A**). SW1116 has reduced proliferation and migration significantly in the presence of DLC1 overexpression (**Figures 1B, C**).

DLC1 was found to repress PTHLH transcription and secretion and eventually lead to decrease breast cancer bone metastasis (7). We were wondering if DLC1 promoted the secretion of certain factors to block cell migration. We collected and concentrated DLC1-overexpressed SW1116 cell CM to culture wild-type colon adenocarcinoma cell lines SW1116 and HCT116 (**Figure 1D**). Surprisingly, compared with the cells cultured by empty vector control CM, the cells cultured by DLC1-overexpressed CM showed a significant reduction in cell migration (**Figures 1E, G**). However, the same effect was not observed in cell proliferation assay (**Figures 1F, H**). The results above strongly suggested that certain factors of CM resulted in changes in the migration ability of colon cells.

### DLC1 increased MANF secretion

**Figure 2:**
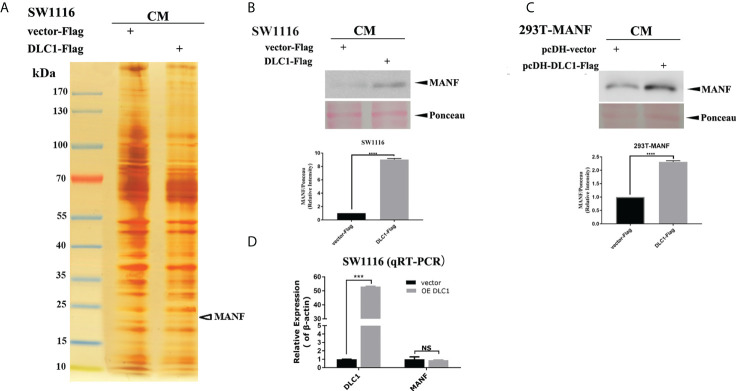
DLC1 promoted the MANF secretion. **(A)** MANF was identified by mass spectrometry from the SW1116 cells cultured by overexpressed DLC1 CM. **(B, C)** Western blot showed that MANF protein expression increased in CM of stable DLC1-expressed SW1116 and HEK-293T compared with the vector group. The bottom panel shows the relative intensity of MANF expression compared with ponceau for the upper panel Western blot result. **(D)** MANF mRNA level was not altered by DLC1 overexpression in SW1116 as determined by qRT-PCR. ***p<0.001, ****p<0.0001, compared with vector group. NS, no significant difference compared with the vector group.

To figure out certain factors, overexpressed DLC1 SW1116 CM was concentrated and separated on an SDS-PAGE gel and then silver stained. The specific band around 20 kDa was analyzed and identified as MANF by quantitative mass spectrometry (MS) (**Figure 2A**). We validated the result in stably DLC1-expressed SW1116 and HEK-293T cells. MANF protein levels in these two CMs were upregulated significantly (**Figures 2B, C** and **S3**). However, MANF mRNA level was not affected by overexpressed DLC1 (**Figure 2D**). From the above results, it could be concluded that DLC1 enhanced MANF delivery in different cells, but did not influence transcription of MANF.

### DLC1 depended on MANF to inhibit colon adenocarcinoma cell migration

**Figure 3:**
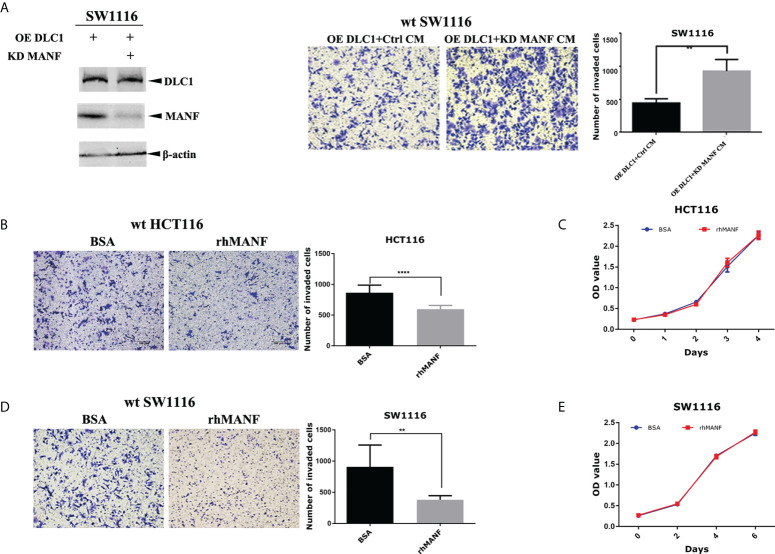
DLC1 inhibited colon adenocarcinoma cell migration depending on MANF. **(A)** We knocked down MANF expression by using specific shRNA in stable DLC1-expressed SW1116 cells. It was verified by Western blot. The ability of these cells to migrate increased as the MANF expression decreased. The concentration of CM added in the upper chamber was 1 μg/ml. **p < 0.01, compared with the vector group. **(B–E)** In comparison with negative control (same concentration of BSA), wild-type SW1116 and HCT116 treated with 200 ng/ml recombinant MANF decreased the ability to migrate, but recombinant MANF had no effect on proliferation. **p < 0.01, ****p < 0.0001 compared with the vector group.

To confirm the effect of MANF on colon cells, we knocked down MANF expression by using specific short hairpin RNA (shRNA) in stably DLC1-expressed SW1116 cells (**Table 1**). The ability of the cell migration increased as the MANF expression decreased (**Figure 3A**). It implied that MANF was the crucial molecular for DLC1 to inhibit cell migration. Wild-type SW1116 and HCT116 were treated with recombinant MANF protein. In comparison with the negative control (same concentration of BSA), the two colon adenocarcinoma cells treated with 200 ng/ml MANF decreased their ability to migrate, but there was no influence on proliferation (**Figures 3B**–**E**). The results, which were in accordance with the ones treated with CM, indicated that MANF was the key factor for inhibition of colon adenocarcinoma cell migration.

### DLC1 affected the interaction of GRP78 and MANF

**Figure 4:**
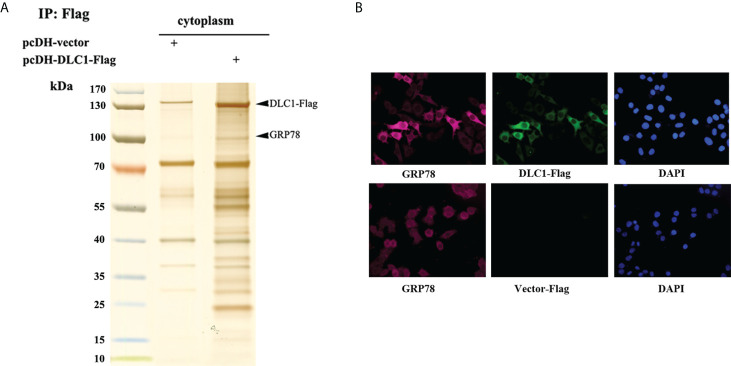
DLC1 co-localized with GRP78 in cytoplasm. **(A)** GRP78 was identified in cytoplasmic extracts of HEK-293T stably DLC1-Flag expressed group. **(B)** Immunofluorescence assay showed exogenous DLC1-Flag and endogenous GRP78 co-localized in cytoplasm of SW1116.

In order to find out what triggered DLC1-mediated MANF release, we collected cytoplasmic extracts of HEK-293T stably expressed DLC1-Flag and subjected them to immunoprecipitation (IP) with anti-Flag M2 magnetic beads. The specific band of glucose-regulated protein 78 kDa (GRP78) was identified *via* MS (**Figure 4A**). The further indirect IF assays verified by transfected DLC1-Flag and endogenous GRP78 were co-localized in the cytoplasm of SW1116 (**Figure 4B**). GRP78, the ER-resident chaperone, interacted with MANF directly and retained MANF in ER instead of secretion (16). We wondered if interaction between DLC1 and GRP78 influenced retention of MANF.

**Figure 5:**
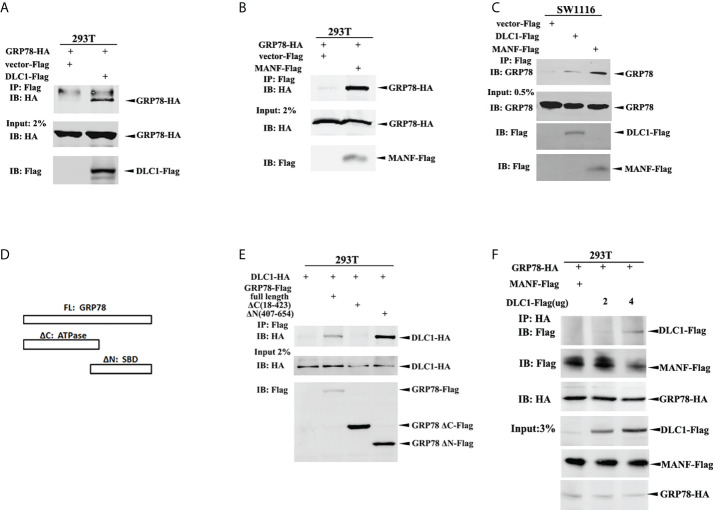
DLC1 competed binding to GRP78 to release MANF. **(A)** GRP78-HA interacted with DLC1-Flag in HEK-293T cells. **(B)** GRP78-HA interacted with Flag-MANF in HEK-293T cells. **(C)** The exogenous DLC1-Flag or MANF-Flag interacted with endogenous GRP78 in colon cells SW1116. **(D)** We constructed truncations of GRP78 by domains (ATPase domain and SBD domain) and tagged with Flag to participate in Co-IP. **(E)** The ΔN truncation (SBD domain) and full length of GPR78 interacted with DLC1 in HEK-293T cells. **(F)** Increasing amount of DLC1-Flag gradually abolished the interaction between GRP78-HA and MANF-Flag.

We validated interactions through co-immunoprecipitation (IP) assay. Accordingly, HEK-293T cells were transfected with constructs encoding GRP78-HA and DLC1-Flag or Flag-MANF; the whole cell lysate was subjected to IP with antibody against Flag, followed by immunoblotting (IB) with antibody HA. The extracts from IP assay contained GRP78-HA as expected (**Figures 5A, B**). Also, exogenous DLC1-Flag or Flag-MANF was confirmed to interact with endogenous GRP78 in colon cell SW1116 (**Figure 5C**). Furthermore, we constructed truncations of GRP78 according to different domains (17) (**Figure 5D**) and tagged with Flag to participate in Co-IP with HA-DLC1. It turned out that the ΔN truncation (SBD) and the full length of GPR78 interacted with DLC1 (**Figure 5E**). However, recent research using bio-layer interferometry reported that MANF associated with ΔC truncation of GRP78 (18), which is not the same binding domain of DLC1.

In order to clarify the effect of these three proteins, we then transfected GRP78-HA with both DLC1-Flag and Flag-MANF into HEK-293T cells, and found that an increasing amount of DLC1-Flag gradually abrogated the interaction between GRP78-HA and Flag-MANF (**Figure 5F**). Taken together, although DLC1 and MANF did not share the same binding site of GRP78, DLC1 did affect interaction between GRP78 and MANF.

## Discussion

**Figure 6:**
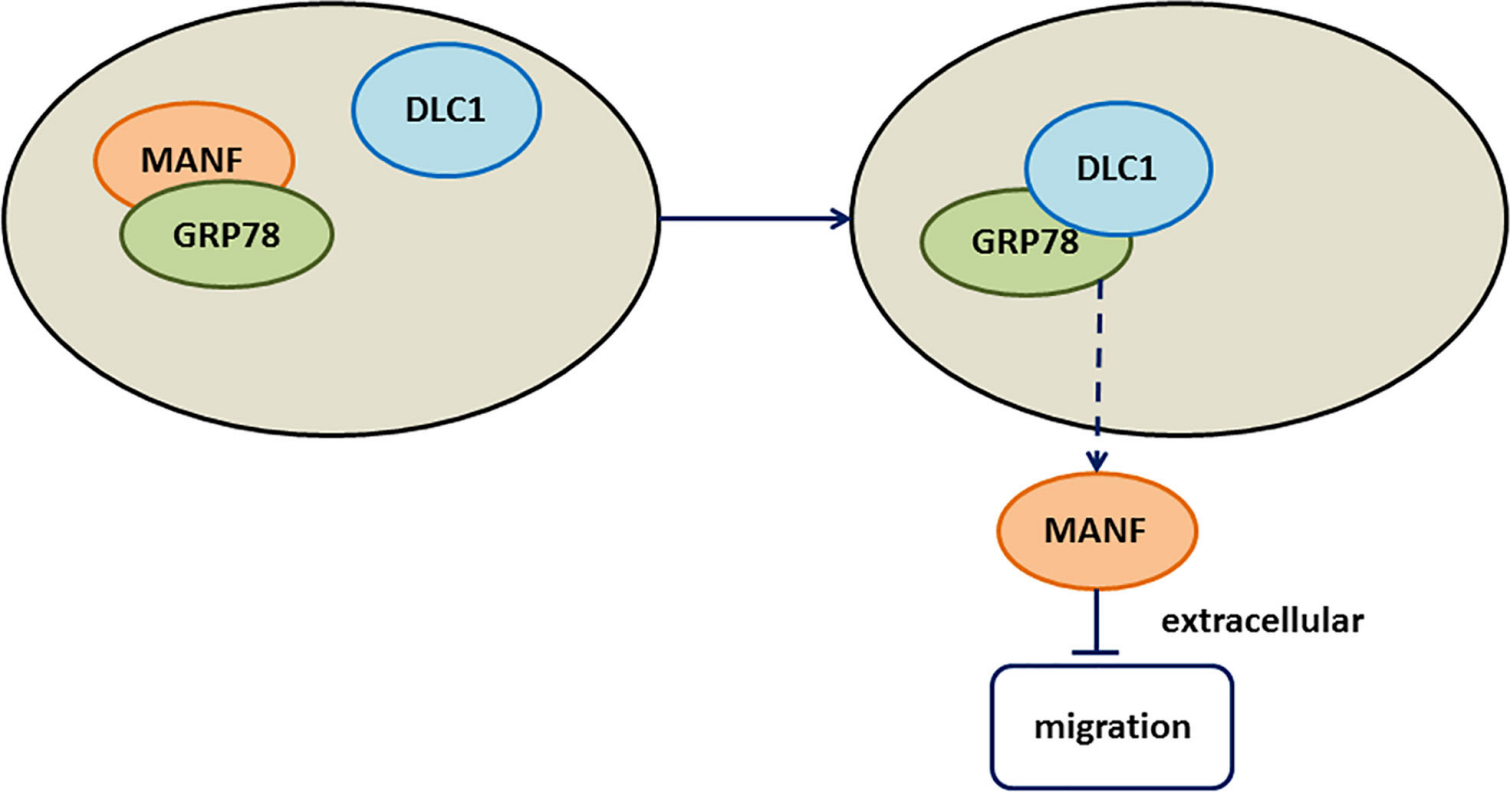
Working model. The working model showed that DLC1 interacted with GRP78 to increase the release of MANF, which inhibited wild-type colon cell migration.

Here, we found that DLC1 was downregulated in CRC and overexpressed DLC1 promoted the secretion of MANF to inhibit colon adenocarcinoma cell migration. Mechanistically, DLC1 affected MANF retention in ER by abrogating the interaction between MANF and GRP78 (**Figure 6**).

DLC1 is mapped to the chromosome 8p21-22 region that is deleted in various human cancers commonly and is assumed to harbor tumor suppressor genes (19). Undetectable or reduced levels of DLC1 are observed in 70% of colon cell lines (19), whereas the promoter methylation of DLC1, relating to decreased gene expression and advanced Duke’s stages, is seen in 60% of CRC tissues in our previous study (20). It encodes a RhoGAP protein to inactivate Rho and Cdc42, resulting in the inhibition of malignant cell proliferation and migration in a GAP-dependent and cytoskeleton-dependent manner (3). On the other hand, there are accumulating proofs that DLC1 plays its role of interacting with some partners, such as PTEN, Talin, FAK, Tension, and EF1A1, in a GAP-independent manner (5, 21–24). Also, DLC1 suppressing PTHLH transcription and secretion uncovers the possibility of using its functions in a cytoskeleton-independent manner (7). In this study, DLC1-overexpressed CM was proved to reduce the migration of wild-type colon cells, but had no impact on proliferation. We speculated that some certain factor in CM only worked on part of the DLC1 utility. To figure out the particular factor, the CM samples were concentrated and separated by SDS-PAGE gel, and some specific bands stood out with the help of silver stain. One protein in these bands was identified by MS as the secretory protein, MANF.

MANF, also named arginine-rich mutated in early tumors (ARMET) or arginine-rich protein (ARP), is found to be mutated in different human solid tumors, including lung, breast, prostate, pancreatic, and renal cancer in the 1990s (25–27). However, all these mutations are verified as normal polymorphisms (28). The research on MANF and cancers has been silent for quite a long time. Still, the recent study on MANF associating ER stress and inflammation in hepatocellular carcinoma brings the attention back to cancer again (10). In our study, we found that DLC1 enhanced MANF delivery, but not its transcription. Furthermore, secretory MANF blocked CRC migration and thereby enriched MANF function in cancer. However, the key question was how DLC1 released MANF.

The secretion of MANF is modulated *via* KDEL receptors (29) and GRP78 (30). GRP78, as a molecular chaperone in ER, is involved in correct protein folding and misfolded protein degradation. Gene mutations and genomic rearrangements result in cancers and also produce a pile of misfolded proteins. This is the reason for GRP78 being frequently triggered in cancers (31). Many studies demonstrate that GRP78 plays a major role in tumor cell proliferation, angiogenesis, metastasis, and drug resistance (32). GRP78 contained an ATP binding domain and a peptide substrate binding domain (SBD) (**Figure 5B**). It is reported that MANF interacted with the NBD of ADP-bound GRP78 (18), which resulted in MANF retention in ER and decrease of secretion (16). We found that GRP78 formed a complex with DLC1 or MANF, respectively. Although DLC1 and MANF did not share the same binding site of GRP78 (18), DLC1 still abolished the interaction between GRP78 and MANF, and eventually decreased MANF retention and increased its secretion. We inferred that the mutual action between DLC1 and GRP78 might change the conformation of GRP78 or altered ADP-bound GRP78 to the ATP-bound state, which affected MANF binding (18). However, the mechanism remains to be further studied.

## Conclusion

In summary, the present study showed that DLC1 interacted with GRP78 to increase the release of MANF, which inhibited the migration of wild-type colon adenocarcinoma cells. Our data could enrich the function of exogenous MANF in cancers, and MANF might act as a small molecule for the treatment of CRC.

## Data availability statement

The original contributions presented in the study are included in the article/**Supplementary Material**. Further inquiries can be directed to the corresponding authors.

## Author contributions

Conceptualization, HXP; Methodology, YMC, YX and XQZ; Investigation, YMC, YX, XQZ and YTH; Resources, YMC, YX, YLD, FLZ and JL; Software, YMC and YX, Funding Acquisition, HXP, YMC and YX; Writing- Original Draft, YMC; Writing-Review & Editing HXP and DMY. All authors contributed to the article and approved the submitted version.

## Funding

This work was supported by Interdisciplinary Program of Shanghai Jiao Tong University (No. ZH2018QNB24), and Shanghai Municipal Health Commission Key Laboratory of Gastrointestinal Tumor Innovation and Translation (No. ZDSYS-2021-01 ), and Science and Technology Commission of Changning District, Shanghai (No. CNKW2018Y02), and Natural Science Foundation of Shanghai (No. 18ZR1434900), and Foundation of Shanghai Tongren Hospital Rising Star (TRKYRC-xx202211).

## Conflict of interest

The authors declare that the research was conducted in the absence of any commercial or financial relationships that could be construed as a potential conflict of interest.

## Publisher’s note

All claims expressed in this article are solely those of the authors and do not necessarily represent those of their affiliated organizations, or those of the publisher, the editors and the reviewers. Any product that may be evaluated in this article, or claim that may be made by its manufacturer, is not guaranteed or endorsed by the publisher.
