# Supplementary material for: DLC1 inhibits colon adenocarcinoma cell migration by promoting secretion of the neurotrophic factor MANF
Source: Front Oncol. 2022 Sep 14;12:900166. doi: 10.3389/fonc.2022.900166 (PMC9515611; doi:10.3389/fonc.2022.900166)
Supplement: Supplementary file 1 [file DataSheet_1.docx]

**Supplemental Files**

**Fig S1: DLC1** **was down-regulated in tumor tissues of CRC.**


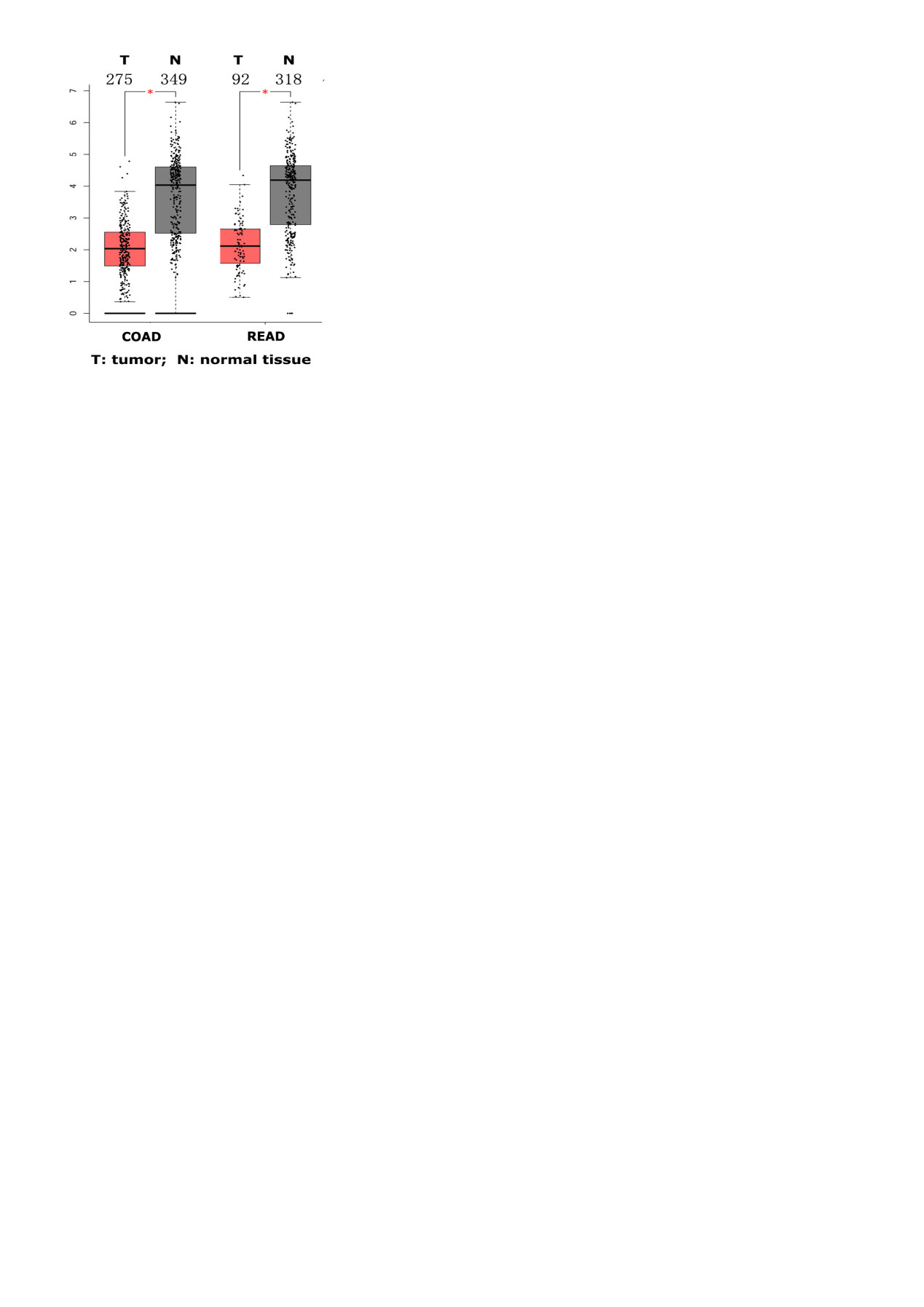


DLC1 expression was down-regulated in tumor tissues (n=275, 92) compared with normal ones (n=349, 318) from an analysis of colon adenomas (COAD) and rectal adenomas (READ) in The Cancer Genome Atlas (TCGA) database *(*[*http://gepia.cancer-pku.cn/detail.php*](http://gepia.cancer-pku.cn/detail.php)*)*. *P<0.05, compared with normal tissues.

**Fig S2: DLC1 expression was lower in metastasis cells.**


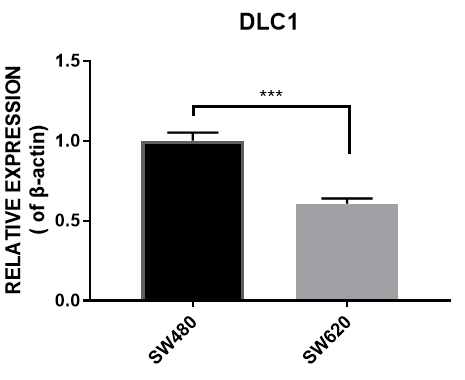
These two cells derived from the same manner. SW480 was initiated from the primary adenocarcinoma, while SW620 was from a lymph node. We found the DLC1 expression was lower in metastasis cells (SW620). ***P<0.001, compared with SW480.

**Fig S3: DLC1 promoted the MANF secretion in SW1116.**





MANF protein expression in CM of DLC1-expressed SW1116 compared with vector group as determined by ELISA. *P<0.05, **P<0.01, ***P<0.001, compared with vector group.
